# Hepatitis B Vaccination Coverage and Its Determinants Among Healthcare Personnel in Chengalpattu District: A Cross-Sectional Study

**DOI:** 10.7759/cureus.106638

**Published:** 2026-04-08

**Authors:** Anushuya M., Raja D., Vijayalakshmi S., Meena D., Priyanka Ramesh

**Affiliations:** 1 Community Medicine, Chettinad Hospital and Research Institute, Chettinad Academy of Research and Education, Chennai, IND

**Keywords:** healthcare personnel, hepatitis b, occupational exposure, vaccination coverage, vaccine uptake

## Abstract

Background: Hepatitis B virus (HBV) is a major occupational health hazard among healthcare personnel (HCP). In India, 40 million people are chronically affected despite the availability of effective vaccinations. Hence, this study focuses on assessing the vaccination coverage and identifying the determinants of hepatitis B vaccine uptake among HCPs in South India.

Objective: To estimate the Hepatitis B vaccination coverage and identify factors associated with Hepatitis B vaccination uptake among HCP in Chengalpattu district.

Methodology: A cross-sectional study was conducted over a period of three months at primary healthcare institutions in the Chengalpattu district. A total of 270 HCP were selected through stratified random sampling, and data were collected using a semi-structured questionnaire after reviewing similar work in the literature. The collected data were entered into MS Excel and analyzed using SPSS version 21 (IBM Corp., Armonk, NY). Logistic regression analysis was used to test the factors influencing Hepatitis B vaccination, and a *P*-value <0.05 was considered significant.

Results: A total of 270 HCP participated in the study. The mean age ± standard deviation (SD) of the HCP was 35.78 ± 10.8. Most participants were between 21 and 40 years old (186, 68.9%). About 227 (84.1%) were females. Of the study participants, 98 (36.6%) had received complete Hepatitis B vaccination (three doses), of whom 12 (4.4%) had also received complete vaccination along with booster doses. Multivariable logistic regression analysis identified marital status (adjusted odds ratio (AOR) = 0.413; 95% confidence interval (CI): 0.234-0.730; *P* = 0.002) and educational status (AOR = 2.638; 95% CI 1.162-5.985; *P* = 0.020) as factors associated with complete hepatitis B vaccination among HCP.

Conclusions: In this study, only one-third of the participants were completely vaccinated against hepatitis B. Thus, improving targeted educational interventions, access to vaccination services, and strengthening institutional policies will improve vaccination coverage among HCP.

## Introduction

Hepatitis B is a dynamic, multi-modal, highly contagious, and pathogenic illness primarily affecting the human liver and caused by the hepatitis B virus (HBV) [1]. HBV is a well-recognized occupational hazard for healthcare personnel (HCP) because of frequent exposure to patients’ blood and body fluids during routine clinical care; HCP in highly endemic settings face particularly elevated risk [2]. Hepatitis B vaccination produces more than 90% of antibody response after the recommended three-dose series at 0, 1, and 6 months [3]. Studies from India report suboptimal HBV vaccination uptake among HCP, with coverage estimates in many center-based surveys ranging between 25% and 60% across various settings, indicating gaps in vaccine delivery and uptake [4,5].

HCP in India often show suboptimal hepatitis B vaccination coverage and waning immunity over time. A study of 2,162 HCWs in India found that only 55.4% had been vaccinated, while 28% were completely unvaccinated and 17% unaware of their vaccination status [6]. Another cross-sectional study from Tamil Nadu 80.5% of the healthcare workers (HCWs) had complete hepatitis B vaccine,18.5% were partially vaccinated, and the remaining 0.9% were unvaccinated [7]. A study from Tamil Nadu showed that 44.2% of the HCWs had complete hepatitis B vaccination, while 56.8% were either partially vaccinated or unvaccinated; 28.4% were unvaccinated [8]. These findings highlight the need for context-specific assessments to understand vaccination coverage and the factors influencing vaccine uptake among HCP in different healthcare settings. This study aimed to assess hepatitis B vaccination coverage and identify factors that influence vaccination among HCP, which may help strengthen vaccination campaigns, boost immunization, and reduce the risk of hepatitis B transmission in healthcare settings. The objective was to estimate the hepatitis B vaccination coverage and identify factors associated with hepatitis B vaccination uptake among HCP in Chengalpattu district.

## Materials and methods

Study design, setting, and duration

This cross-sectional study was conducted from September 2025 to November 2025, among 270 HCP in Block Primary Health Centers (PHCs)/Community Health Center (CHC), and PHC of the Chengalpattu district, Tamil Nadu, India.

Inclusion and exclusion criteria

HCP aged 21 years and above were included, while participants who were uncooperative and those who were on extended leave were excluded from the study.

Sample size calculation

A sample size of 270 was determined based on a study by Mohanty et al., where *p* = 0.19 [9].

The sample size for estimating the population proportion was calculated using the formula:

\[ n = \frac{Z^2\, p (1 - p)}{d^2} \]

where *n* is the sample size, *Z* is the statistic corresponding to the level of confidence, *p* is the expected prevalence, *q* = (1 - *p*), and *d* is the precision. Assuming *Z* = 95% confidence interval (CI), prevalence *p* = 0.19, *q* = 0.81, and *d* = 0.05 (allowable error of 5%), the sample size was calculated to be 246. Accounting for a 10% non-response rate, the final sample size was derived to be 270.

Sampling technique

A multistage stratified random sampling technique was employed. In the first stage, all government primary healthcare facilities in Chengalpattu district were stratified into three levels: Block PHCs, CHCs, and PHCs. In the second stage, all eight Block PHCs were included (census). From each block, one CHC was selected using simple random sampling, resulting in eight CHCs. Additionally, 22 PHCs were selected from the list of all PHCs using simple random sampling. In the third stage, the required sample size (*n* = 270) was allocated to each selected facility using probability proportional to size (PPS), based on the number of HCWs available at each facility. In the final stage, individual HCWs were selected from each facility using simple random sampling from the staff registers.

Data collection and statistical analysis

Permission to conduct the study was obtained from the director of Public Health and the District Health Officer of Chengalpattu district. The self-reported data were collected using a standardized semi-structured questionnaire that was developed through a systematic process. The questionnaire was formulated based on an extensive literature review of similar studies on hepatitis B vaccination among HCWs. It was then reviewed by subject matter experts in community medicine to ensure content validity. A pilot study was conducted among 27 participants (10% of the final sample size) using the semi-structured questionnaire among HCP of Chengalpattu district to assess clarity, comprehensibility and feasibility. Based on the responses and feedback from the pilot study, necessary modifications were made to the questionnaire. The internal validity of the questionnaire was analyzed using reliability analysis. The Cronbach’s alpha coefficient of the questionnaire was 0.80, which indicated good internal consistency and reliability of the instrument. The final validated questionnaire was then used for data collection (Appendix). The outcomes were documented in a Microsoft Excel Spreadsheet and SPSS, version 21 (IBM Corp., Armonk, NY).

The questionnaire had the following sections: (A) HBV vaccination status: non-vaccination, partial vaccination (1-2 doses), and complete vaccination (3 doses with booster dose); and (B) factors influencing hepatitis B vaccination uptake.

The qualitative variables were described in terms of frequencies and percentages. Continuous variables were categorized for the purpose of statistical analysis. Using the median age of the population, age was categorized into younger (21-40 years) and older HCP (41-60 years). Work experience was categorized into three categories of five years interval: 1-5, 6-10, and >10 years. The inferential statistics were carried out through Pearson’s chi-square test to determine the association between independent variables and hepatitis B vaccine uptake among HCP. Those variables that had significance were subjected to perform bivariate logistic regression to obtain crude odds ratio (COR) and 95% CIs. Further, multivariate analysis was then performed to obtain adjusted odds ratio (AOR) and 95% CI. *P*-value <0.05 was considered significant.

For logistic regression analysis, selected categorical variables (education and profession) were regrouped into two categories to ensure adequate cell size and statistical stability of the model. Educational status was dichotomized into schooling-level education (primary and secondary and higher secondary) and higher education (diploma and degree or above). Profession was categorized into clinical HCP (medical doctors, nurses, and laboratory technicians) and support staff (biomedical waste handlers).

The data were checked completely after data collection. Those questionnaires that had incomplete details were excluded. Hence, participants with all the available data were included in the analysis. No subgroup analysis was performed.

Ethical consideration

Approval from the Chettinad Hospital and Research Institute IHEC (Institutional Human Ethics Committee) (Ref. No.: IHEC-II/1015/26) was obtained before the commencement of the study. All HCP were provided with detailed information about the study including its benefits, procedures and confidentiality of their data in both local and English languages. Participants who needed guidance regarding vaccination status and occupational health risks were guided and encouraged to seek further medical advice when necessary. Ethical considerations were held up throughout the research process to ensure privacy and confidentiality of the participants.

Variables and measurement

The primary outcome of the study on complete hepatitis B vaccination was defined as receiving three doses of hepatitis B vaccine, with or without a booster dose. The sociodemographic factors such as age, gender, marital status, education, profession and work experience were considered as predictors of vaccination uptake, while marital status, educational status and profession were considered as confounding variables. Selection bias was minimized through stratified sampling. Confidentiality was ensured to reduce social desirability bias regarding vaccination status and reasons for non-vaccination.

## Results

All 270 healthcare personnel agreed to participate, resulting in a response rate of 100%. Table 1 shows the baseline characteristics of study participants. Of the 270 participants, 15.9 were males and 84.1% were females. The mean ± SD age of the participants was 35.78 ± 10.8 years. The majority of respondents had completed diploma (98, 36.3%) and degree or higher qualification (110, 40.7%). Nurses constituted the largest professional group (161, 59.6%), followed by medical doctors (57, 21.1%), with lab technicians and biomedical waste workers each constituting 26 (9.6%). More than half of the participants (136, 50.4%) had one to five years of work experience, indicating young HCP.

**Table 1:** Sociodemographic profile of study participants (N = 270). Values are expressed as frequency and percentage.

Variable	Categories	Frequency (*n*)	Percentage (%)
Age (years)	21-40	186	68.9
	41-60	84	31.1
Gender	Female	227	84.1
	Male	43	15.9
Marital status	Married	199	73.7
	Unmarried	71	26.3
Educational status	Primary	19	7.0
	Secondary and higher secondary	43	15.9
	Diploma	98	36.3
	Degree and above	110	40.7
Profession	Medical doctors	57	21.1
	Nurses	161	59.6
	Lab technician	26	9.6
	Biomedical waste workers	26	9.6
Work experience	1-5 years	136	50.4
	6-10 years	85	31.5
	>10 years	49	18.1

Table 2 describes the HBV vaccination status among the selected HCP. Only one-third (98, 36.3%) of the participants were fully vaccinated with three doses as recommended by the vaccine schedule, out of which 12 (4.4%) had received a booster dose. Nearly half (137, 50.7%) were partially vaccinated with one or two doses, and 35 (13.0%) were unvaccinated, indicating incomplete protection. Among 172 (63.7%) unvaccinated and partially vaccinated participants, 74 (43%) had missed the dose, 63 (36.6%) had a misbelief that a single dose provides sufficient immunity, 16 (9.3%) had perceived low risk, while 12 (7%) had intentionally ignored the vaccine, and 7 (4.1%) had poor awareness, indicating gaps in knowledge and risk perception. Similarly, among completely vaccinated participants, 76 (77.6%) had fear of developing the disease and 22 (22.4%) believed in vaccine safety and efficacy.

**Table 2:** HBV vaccination status of healthcare workers (N = 270). Values are expressed as frequency and percentage. HBV, hepatitis B virus

Vaccination status	Frequency (*n*)	Percentage (%)
Non-vaccinated	35	13.0
Partially vaccinated (1-2 doses)	137	50.7
Fully vaccinated (3 doses)	86	31.9
Received >3 doses/booster	12	4.4
Reasons for non/partial vaccination (*n* = 172)		
Poor awareness	7	4.1
Ignored the vaccine on purpose	12	7.0
Perceived low risk	16	9.3
Missed the dose	74	43.0
Perceived that a single dose provides immunity	63	36.6
Reason for complete vaccination (three doses and booster doses) (*n* = 98)		
Fear of disease	76	77.6
Belief in vaccine safety and efficacy	22	22.4

Table 3 compares the demographic profile and hepatitis B vaccination status of HCP who reported complete vaccination with three doses and a booster dose (*n* = 98) with those who reported being unvaccinated or partially vaccinated (*n* = 172). The results show that age, gender, and work experience were not significantly associated with hepatitis B vaccination status, as indicated by *P*-value > 0.05.

**Table 3:** Factors influencing poor hepatitis B vaccine coverage, N = 270. Chi-square test applied. *Statistically significant (*P* < 0.05).

Variable	Categories	Hepatitis B vaccination status		Chi-square	*P*-value
		Unvaccinated and partially vaccinated, No (*n *= 172)	Complete vaccination (three and booster doses), Yes (*n* = 98)		
Age (years)	21-40	116 (62.4%)	70 (37.6%)	0.463	0.496
	41-60	56 (66.7%)	28 (33.3%)		
Gender	Female	148 (65.2%)	79 (34.8%)	1.377	0.241
	Male	24 (55.8%)	19 (44.2%)		
Marital status	Married	139 (69.8%)	60 (30.2%)	12.361	<0.001*
	Unmarried	33 (46.5%)	38 (53.5%)		
Educational status	Primary	12 (63.2%)	7 (36.8%)	18.331	<0.001*
	Secondary and higher secondary	38 (88.4%)	5 (11.6%)		
	Diploma	65 (66.3%)	33 (33.7%)		
	Degree and above	57 (51.8%)	53 (48.2%)		
Profession	Medical doctors	19 (33.3%)	38 (66.7%)	30.199	<0.001*
	Nurses	115 (71.4%)	46 (28.6%)		
	Lab technician	17 (65.4%)	9 (34.6%)		
	Biomedical waste workers	21 (80.8%)	5 (19.2%)		
Work experience	1-5 years	80 (58.8%)	56 (41.2%)	3.079	0.214
	5-10 years	57 (67.1%)	28 (32.9%)		
	>10 years	35 (71.4%)	14 (28.6%)		

In contrast, marital status (*P *< 0.001), educational status (*P* < 0.001), and profession (*P *< 0.001) showed statistically significant association with hepatitis B vaccination status. The findings indicate that improving overall hepatitis B vaccination coverage in healthcare facilities helps to reduce the occupational risk among HCP.

Table 4 presents the logistic regression analysis of factors influencing hepatitis B unvaccination and partial vaccination among HCP. In the unadjusted analysis, married HCP had significantly higher odds of being unvaccinated or partially vaccinated compared to unmarried participants (unadjusted odds ratio (UOR) = 0.375; 95% CI: 0.215-0.654; *P* < 0.001). This association remained statistically significant after adjusting for potential confounders (AOR = 0.413; 95% CI: 0.234-0.730; *P* = 0.002).

**Table 4:** Logistic regression analysis of factors influencing poor hepatitis B vaccine coverage, N = 270. Those variables that were associated with hepatitis B vaccination in the chi-square test were subjected to bivariate and multivariate logistic regression analysis. *Statistically significant (*P *< 0.05). LT, laboratory technician

Variables	Categories	Unvaccinated and partially vaccinated, No (*n *= 172)	Complete vaccination (three and booster doses), Yes (*n *= 98)	Unadjusted odds ratio	95% CI	*P*-value	Adjusted odds ratio	95% CI	*P*-value
Marital status	Unmarried	33 (46.5)	38 (53.5)	0.375	0.215-0.654	<0.001*	0.413	0.234-0.730	0.002*
	Married	139 (69.8)	60 (30.2)	1			1		
Educational status	School-level education	50 (80.6)	12 (19.4)	2.937	1.476-5.843	0.002*	2.638	1.162-5.985	0.020*
	Degree and diploma	122 (58.7)	86 (41.3)	1			1		
Profession	Biomedical waste handlers	21 (80.8)	5 (19.2)	2.587	0.943-7.095	0.065	0.987	0.292-3.280	0.972
	Doctors, nurses, and LTs	151 (61.9)	93 (38.1)	1			1		

Educational status was significantly associated with vaccination status. HCP with schooling-level education had higher odds of being unvaccinated or partially vaccinated compared to those with diploma or degree-level education in the unadjusted analysis (UOR = 2.937; 95% CI: 1.476-5.843; *P* = 0.002). This association persisted in the adjusted model (AOR = 2.638; 95% CI: 1.162-5.985; *P* = 0.020), indicating that lower educational status independently increased the likelihood of incomplete vaccination.

Profession did not show a statistically significant association with unvaccinated or partially vaccinated status. Compared to biomedical waste handlers, doctors, nurses, and laboratory technicians did not differ significantly in either the unadjusted (UOR = 2.587; 95% CI: 0.943-7.095; *P* = 0.065) or adjusted analysis (AOR = 0.987; 95% CI: 0.292-3.280; *P* = 0.972).

## Discussion

The present study assessed hepatitis B vaccination coverage and determinants among HCP in primary-level health facilities. The findings suggest that complete vaccination uptake remains suboptimal despite occupational risk. An association was observed between vaccination status and selected sociodemographic characteristics, indicating that individual and professional factors influence vaccine completion.

The vaccination coverage of 36.6% observed in the study is lower than the World Health Organization recommendation for universal hepatitis B vaccination among HCWs. A 2020 meta-analysis by Awoke et al. revealed a pooled hepatitis B vaccination coverage of 20.4% of HCWs in Ethiopia, with significant heterogeneity across countries and healthcare settings [10].

The findings align with evidence synthesized in the systematic review by Lamichhane et al [11], which highlighted wide variability and overall suboptimal hepatitis B vaccination coverage among healthcare trainees and professionals in South Asia. The review emphasized gaps in awareness, perceived risk, and institutional support as recurring barriers, themes that are reflected in the reasons for incomplete vaccination identified in the present study. The consistency between pooled regional evidence and the current findings underscores the persistence of systemic challenges in achieving universal vaccine coverage among HCP.

Recent evidence from India coincides with these findings. A 2025 cross-sectional study by Kundu et al. among HCWs in Eastern India reported that 48.3% had complete vaccination, with lack of awareness and perceived irrelevance as barriers to vaccination [12]. Similarly, research studies have emphasized that vaccine availability alone is insufficient unless supported by targeted education and workplace policies. The present study reinforces this perspective, as higher educational status was independently associated with vaccine completion, suggesting that knowledge translation plays a crucial role in preventive behavior.

The results are comparable with findings reported by Dangol et al. [13], who documented incomplete vaccination coverage among HCWs in a tertiary cardiac center and identified lack of awareness as a major deterrent. Although various studies in different healthcare settings highlight the vulnerability of HCP across levels of care, they suggest that institutional emphasis on vaccination remains inadequate.

Kundu et al. [12] reported marked disparities in vaccination status between professional categories, driven largely by differences in knowledge and perceived relevance of hepatitis B prevention. While profession showed an association in univariate analysis in the present study, it did not retain significance after adjustment, indicating that education rather than job designation may be the underlying determinant influencing vaccination behavior. A study conducted by Priyadarshi et al. among Indian HCWs found that resources available free of cost, mandatory induction vaccination upon joining, educational interventions, and vaccination camps significantly improved vaccination rates among HCWs [14].

Findings from Ghana by Okwan et al. [15] demonstrated that sociodemographic and workplace-related factors significantly influenced vaccine uptake, reinforcing the role of contextual determinants. In contrast, the present study did not observe an independent association with work pattern, possibly due to uniform exposure risks and similar service conditions across primary care settings. The study by Hussain et al. highlighted that HCWs face barriers including unavailability of the vaccine, high vaccine cost, lack of time, and fear of vaccine side effects, which affect vaccination uptake [16].

The study by Omotowo et al. [17] similarly reported low vaccination uptake among HCWs, with education and professional seniority influencing completion rates. Differences in magnitude and predictors across studies may be attributed to variations in healthcare systems, availability of free vaccination programs, and institutional enforcement of occupational health policies.A systematic review by De Koning et al. demonstrated that improved vaccine uptake and vaccination clinics for hepatitis B vaccination programs increased vaccination rates from 13.9% to 95.8% in India, suggesting that policy-driven approaches substantially improve coverage [18].

A study by Nema et al. identified that a high proportion of participants (40%) reported incomplete vaccination due to lack of awareness and a long time gap in hepatitis B vaccination among HCWs in Central India [19]. The implementation of a tracking system and automated SMS reminders is expected to improve hepatitis B vaccination completion rates. Immunological studies have revealed that protective anti-HBs antibody levels are achieved only after completion of the three-dose schedule in most individuals [20].

This study focuses on HCP in primary-level public health institutions, a group that is often underrepresented in vaccination research. The use of stratified random sampling and regression analysis strengthens the internal validity of the findings. The study also included multiple categories of HCP working in primary healthcare institutions, which provides useful insight into hepatitis B vaccination coverage among frontline public health workers. However, limitations of the study include self-reported vaccination status, which is prone to recall or reporting bias. Owing to its cross-sectional design, the study cannot infer causality between the associated factors and hepatitis B vaccine uptake. Moreover, no serological assessment was carried out to confirm protective antibody levels; hence, coverage estimates relied on participants’ reported vaccination history rather than objective immunological evidence. Future research should consider prospective or interventional designs to evaluate the impact of structured vaccination programs, workplace reminders, and policy-driven mandates on improving vaccine completion.

## Conclusions

The study demonstrates suboptimal hepatitis B vaccination coverage among HCP, with educational status and marital status emerging as key determinants of vaccine uptake. Strengthening institutional vaccination policies and targeted educational interventions may improve vaccine completion and reduce occupational risk among HCP.

## Figures and Tables

**Figure 1:**
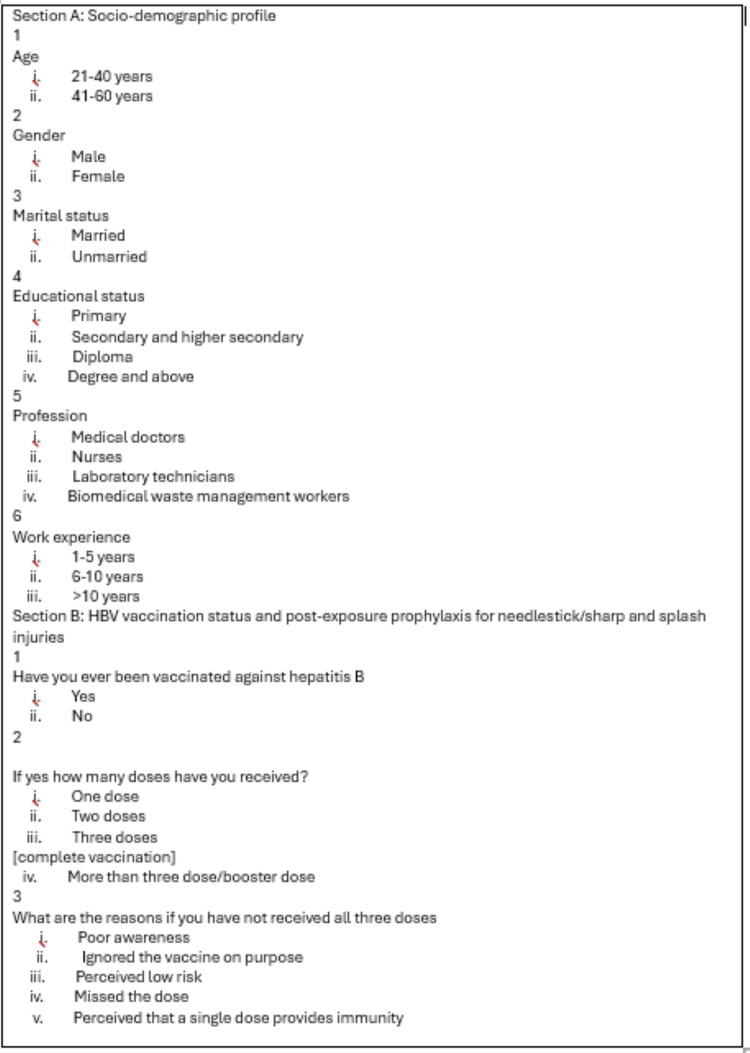
Questionnaire on Hepatitis B vaccination coverage and its determinants among healthcare personnel.

## Appendices

Appendix: Survey questionnaire
